# An evaluation of long-term changes in alcohol use and alcohol problems among clients of the Swedish National Alcohol Helpline

**DOI:** 10.1186/1747-597X-9-22

**Published:** 2014-06-03

**Authors:** Nelleke Heinemans, Mats Toftgård, Kerstin Damström-Thakker, Maria Rosaria Galanti

**Affiliations:** 1 Centre for Epidemiology and Community Medicine, Stockholm County Council, Box 1497, 171 29, Solna, Sweden; 2 Department of Public Health Sciences, Karolinska Institutet, 171 77 Stockholm, Sweden

**Keywords:** Alcohol use disorders, AUDIT, Brief intervention, Motivational Interviewing, Telephone counselling, Gender

## Abstract

**Background:**

The Swedish National Alcohol Helpline was developed with the intention to provide an easily available, low threshold service to hazardous and harmful alcohol users in the community. The primary aim of this study was to describe the 12-month outcome of a cohort of clients and to evaluate whether these varied as a function of the intensity of exposure to the intervention.

**Methods:**

The alcohol use and alcohol problems of a cohort of 191 clients accessing the service between 1 April 2009 and 1 February 2011 were assessed by telephone survey at the time of the first call and after 12 months. Change in AUDIT score between baseline and follow-up was used as primary outcome. Intensity of exposure was defined by number of counselling sessions.

**Results:**

At 12-month follow-up, respondents had significantly reduced their AUDIT score to half of the baseline values, and one third of the participants were abstinent or consumed alcohol at a low-risk level. Participating in more than one counselling session as compared to one session was associated with a tendency to shift to a lower AUDIT zone at follow-up among women.

**Conclusions:**

The Alcohol Helpline provides a viable community service for harmful and hazardous alcohol users. Future randomized studies including other treatment or control conditions are warranted in order to strengthen our preliminary conclusion of possible effectiveness of the counselling provided at the helpline, as well as to explore further the role of gender in moderating the treatment’s effect.

## Background

Most individuals who seek formal treatment for alcohol problems do so at a rather late stage after the onset of alcohol dependence, i.e. after significant psychosocial and medical consequences of their alcohol use have become evident. This delayed help-seeking behaviour is primarily due to a combination of underestimation of risks [1,2], of a lack of motivation [3], of feelings of shame [1,3,4] and of feelings of stigmatisation [2,3]. Therefore, attempts have repeatedly been made to develop interventions suitable to individuals with hazardous or harmful alcohol use, i.e. use involving either a risk for or the actual presence of physical and/or psychological complications [5]. The aim of the intervention is to provide professional support that may help reducing alcohol use, thus decreasing the risk of progression into alcohol dependence. The development of the Swedish Alcohol Helpline represents an attempt to meet these needs and rested on the following combined evidence. First, many studies carried out in health care settings have shown that brief interventions are both effective and cost-effective, especially for patients with hazardous or harmful alcohol use e.g. [6-10]. Participants in these studies are typically non-treatment-seeking primary care patients identified by opportunistic screening. Second, there is evidence that Motivational Interviewing (MI) has been proved effective in reducing alcohol consumption [11]. Further, telephone-based interventions may be effective in the treatment of mental health problems [12] and for smoking cessation [13] and are available at a low cost [12,14].

To our knowledge, no previous studies evaluated telephone counselling as a stand-alone intervention for self-referred hazardous and harmful alcohol users in the community. Instead, telephone counselling has been provided to patients identified by screening when seeking health care for other reasons e.g. [15-17], as part of multicomponent interventions e.g. [16-18], or as part of extensive efforts to encourage patients treated in specialised substance abuse treatment to attend aftercare or continuing care e.g. [19].

In addition there is scanty evidence concerning gender differences in alcohol related outcomes post-interventions. For instance, previous studies on brief interventions have shown inconclusive results regarding gender differences in treatment outcomes [6-8,20,21].

The Swedish National Alcohol Helpline was developed based on the assumption that an easily available and low threshold service would be attractive to hazardous and harmful alcohol users in the community. Since 2007 the Alcohol Helpline provides nation-wide telephone-based counselling primarily based on MI [22,23] and delivered by specially trained counsellors. The service is operated by the Centre for Epidemiology and Community Medicine (CES) in the Stockholm County Council (SCC) and thus not a part of the SCC’s specialised substance abuse treatment system. The Helpline is funded by both the SCC and the Public Health Agency of Sweden, is free of charge and callers can choose to be anonymous.

The current paper reports the results of a study involving a 12-month follow-up of clients who contacted the Helpline for the first time between 1 April 2009 and 1 February 2011. The main alternative hypothesis of this study was that outcomes related to alcohol use and alcohol problems among clients of the Alcohol Helpline improve as a function of the intensity of exposure to the intervention, and that this improvement does not differ between men and women. The focus on gender-specific outcomes was motivated by the large proportion of female clients requesting support at the telephone Helpline. Secondly, we hypothesized that individuals receiving counselling at the Alcohol Helpline would improve their mental and general health, irrespective of improvement in alcohol related outcomes.

## Materials and methods

### The counselling at the Swedish alcohol helpline

The counselling at the Alcohol Helpline is primarily based on Motivational Interviewing (MI) [22,23], combined with elements of Cognitive Behaviour Therapy. Individual medical advice is not provided. The goal of the counselling is to increase clients’ motivation and skills to change their alcohol use and to prevent relapse. The intervention is adapted to the individual client’s desired achievements as well as to the severity of his/her alcohol problems. Counsellors have a background in health care or health promotion. Prior to employment, they participate in a 14-day basic training programme during four months, dealing with treatment of alcohol problems and use of MI. The MI training has a total duration of six days, including 2.5 days of group coaching based on five audio-taped interviews with simulated clients (professional actors). The trainees’ MI-performance is assessed by coding according to the Motivational Interviewing Treatment Integrity (MITI) Code Version 3.1 [24] performed at the Motivational Interviewing Coding Laboratory (MIC Lab) at the Karolinska Institutet in Stockholm [25]. The MI trainers are all members of the Motivational Interviewing Network of Trainers (MINT). Counsellors are closely supervised at the beginning of their employment. In course of employment, the counsellors’ MI-performance is monitored by MITI-coding of audiotaped sessions and group coaching four times a year. General aspects of the counselling are discussed at monthly meetings and recurring training days. The Alcohol Helpline operates on two or three lines simultaneously, during 33 hours a week. All contacts with the callers are registered in a computerized client record subject to rules of confidentiality commonly used within the Swedish health care system. The intervention protocol encourages repeated sessions that build on the outcome of previous sessions. On request, clients may access the same counsellor in subsequent calls. Callers can also choose between a reactive (caller initiated) contact or a proactive service (counsellor calls at an agreed date and time). The Alcohol Use Disorders Identification Test (AUDIT) [5] is used for the assessment of the client’s alcohol use and alcohol problems. Although hazardous and harmful alcohol users [5] constitute the primary target group, many of the clients may already be dependent. Clients needing additional support are referred to other service providers, including primary care, specialised addiction treatment and Alcoholics Anonymous.

### Study sample

**Figure 1:**
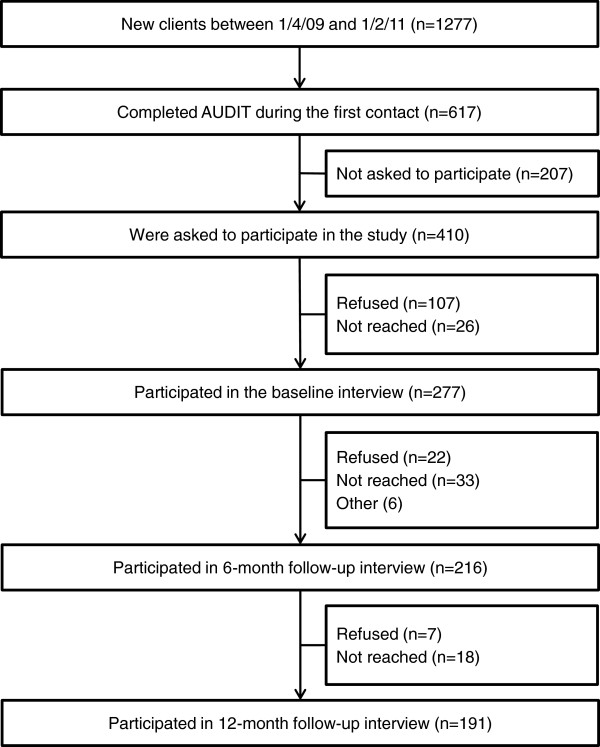
Flow chart.

The present study is based on a 12-month follow-up of a cohort of first-time callers registered between 1 April 2009 and 1 February 2011. In order to have equivalent baseline data for all participants only callers who completed the AUDIT during their first session (n = 617) were eligible for participation in the study. The counsellors were instructed to register whether or not consent was obtained from the eligible clients. They were also required to note whether they did not have the opportunity to ask for consent (for instance because of interrupted conversations or because of the client’s emotional state). Of the 410 clients who were asked to participate 107 (26%) refused participation and 26 (6%) initially consented but could not be reached for the baseline interview. As a result 277 (68%) of the clients that were asked for consent were interviewed at baseline, thus entered the study (Figure 1).The baseline interview was conducted by an independent interviewer (i.e. not a counsellor) within a couple of days following the first call. Clients who participated in the baseline interview were approached for a follow-up survey six months later, and 216 participated. These were approached for the final assessment 12 months after the first call. Seven of them (3%) refused to participate while 18 individuals (8%) could not be reached, leaving 191 individuals (31% of all eligible clients) as participants in the follow-up 12 months after the first session (Figure 1). All interviews were conducted by telephone. Up to ten attempts were made to reach the client for the follow-up interviews.

The study was approved by the Regional Ethics Board at the Karolinska Institutet in Stockholm.

### Data collection

Both the baseline and the follow-up interviews were structured and included standard questions as well as questions designed especially for the purpose of the present study. The baseline interview consisted of 84 questions, covering demographics, alcohol use and alcohol problems, behaviour change goals, previous help seeking for alcohol-related as well as for other health problems, and mental health. At follow-up, questions were added about contacts with the Helpline and other treatment providers since the previous interview. In this study, we used data from the baseline interview and from the 12-month follow-up, because most questions investigating retrospective experiences at baseline used a 12-month frame. In addition to the interview data the AUDIT score at baseline and information on the number and length of telephone sessions with the Helpline were retrieved from the computerized client records.

### Outcome

We defined as primary outcome the change in alcohol use and alcohol problems as assessed by the AUDIT, encompassing ten questions covering alcohol use and alcohol problems during the previous 12 months [5]. This instrument was originally intended for use in primary care, but it has also been widely used in the evaluation of other alcohol-related interventions. The total score varies between 0 and 40, and a variety of cut-points have been discussed [26]. At the time of this study counsellors at the Alcohol Helpline used the following cut-points for different AUDIT zones: “Low risk” (0–7 for men and 0–5 for women), “Hazardous alcohol use” (8–14 for men and 6–12 for women), “Harmful alcohol use” (15–19 for men and 13–19 for women), and “Possible alcohol dependence” (20–40 for both men and women).

The primary outcome variable was defined as downward change of problem severity as defined above, i.e. having shifted to a lower AUDIT zone compared to baseline.

The following secondary outcomes were also considered.

a. Mental health problems were assessed by the Swedish revised version of the screening instrument M.I.N.I. (M.I.N.I. International Neuropsychiatric Interview 5.0.0) [27], intended for use in non-specialised settings. In this study, the screening only covered major depressive episode (MDE, during the two weeks preceding the survey) and generalized anxiety disorder (GAD, during the six months preceding the survey).

b. Self-assessed health, measured by the question (“How would you describe your general health status?”) with five response alternatives (“Very good”, “Good”, “Fair”, “Bad”, “Very bad”).

c. Help seeking for alcohol problems was assessed by asking participants whether they had sought help in health care settings (e.g. general practitioner, specialised addiction care, hospital care), or from other services (e.g. social services or other professional help, self-help programs, support groups). An additional question concerned use of medication for alcohol dependence.

### Treatment predictor

Number of counselling sessions at the Alcohol Helpline was the primary independent variable used to indicate intensity of the intervention. The number of sessions during the 12 months preceding the follow-up assessment was categorized in tertiles of the overall distribution (1 session, 2–3 sessions, 4 or more sessions). The first call to the Alcohol Helpline was considered as a session if it lasted longer than 10 minutes. A subsequent call was considered as a session if it lasted at least five minutes.

### Other covariates

We included the following additional covariates in the analysis, in order to adjust for potential confounding effects of alcohol problem severity, co-morbidity, help seeking and treatment goal.

a. The AUDIT score at baseline as described above.

b. The presence of major depressive episode (MDE) and/or generalized anxiety (GAD) at baseline.

c. Help seeking for alcohol problems during the 12 months preceding each survey.

d. The client’s treatment goal at baseline, assessed by the question: “what is your main goal in changing your alcohol use?” with the following mutually exclusive response alternatives: “to drink smaller amounts of alcohol on each occasion”, “to drink alcohol less often”, “to control drinking”, “to abstain completely from drinking alcohol in the future”, “to abstain from drinking alcohol for a limited period” and “other”.

### Data analysis

Changes in AUDIT score, AUDIT zones and secondary outcomes between baseline and follow-up for the total group of participants were tested with McNemar’s test for binary data, Wilcoxon signed ranks test for ordinal data and paired t-test for interval data.

Ordinal logistic regression was used to estimate the association between number of counselling sessions and AUDIT zones at follow-up (abstinence or low-risk, hazardous alcohol use, harmful alcohol use and alcohol dependence), controlling for AUDIT score, mental health problems and treatment goal (abstinence or controlled drinking) at baseline and help seeking for alcohol problems during the previous 12 months (yes/no). As estimate of association we calculated the Odds Ratios (OR) and corresponding 95% confidence intervals. The test of parallel lines was used to see whether the assumption of proportional odds was met. In case of a rejection of the proportional odds assumption, separate logistic regressions were used to examine how the ORs varied at the different thresholds.

Analyses were carried out using information from participants with complete data, in the following referred to as the analytical sample. To explore the possible modification by gender we tested for interaction effect by adding an interaction term for the number of counselling sessions and gender to the model. In a similar way, we tested for the possible modification by help seeking from other health care providers during the study period. Since there is evidence that individuals with alcohol problems who achieve complete abstinence differ from those who progress to controlled drinking concerning previous alcohol use [28,29], treatment history [28], social environment, stage of change, expectancies and health status [29] the analyses were repeated after exclusion of individuals who were abstinent at follow-up (n = 12 men and n = 8 women).

Analyses were performed using the statistical program package IBM SPSS Statistics (version 20 for Windows, 2011, SPSS Inc., Chicago, IL).

## Results

There were only minor differences between the analytical sample and the eligible clients who did not participate in the 12-month follow-up. The proportion of women (38% and 41% respectively), and the average AUDIT score among women (21 and 22 respectively) were slightly lower among the participants than among the non-participants, while the average age (47 and 43 years respectively) was slightly higher among the participants than among the non-participants.

Table 1 presents the socio-demographic characteristics of the analytical sample at baseline. A majority of the individuals in the sample lived together with their family, were either employed or studying and knew someone who could provide social support in case of personal crisis (Table 1).

**Table 1:** Sociodemographic characteristics of the sample at baseline, by gender

	**Men (n = 117)**	**Women (n = 74)**	**Total sample (n = 191)**
Age (Mean [sd] )	47.0 (13.6)	48.1 (14.3)	47.4 (13.9)
Living arrangements (%)		*	
Married/cohabiting with children	35	16	28
Married/cohabiting without children	29	20	26
Single parent	5	14	8
Living alone	27	49	36
Living with others	3	1	3
Working/studying (%)	74	72	73
Has always someone who can provide social support when in personal crisis (%)	66	65	66

Note: *statistically significant gender difference p < 0.005.

**Figure 2:**
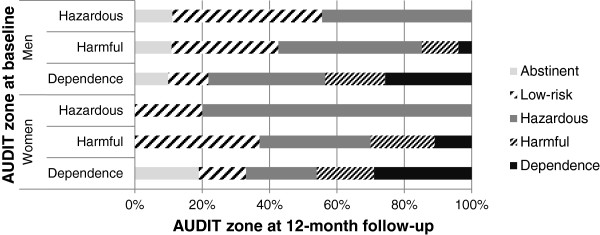
Transition in AUDIT zones between baseline and 12-month follow-up.

In Table 2 selected clinical characteristics of the analytical sample at baseline and at follow-up are presented separately by gender. Based on their AUDIT score 68 percent of the men and 57 percent of the women met the criteria for alcohol dependence at baseline while the others were either hazardous or harmful alcohol users. The mean AUDIT score was almost as high among women as among men (21 and 23 respectively, t (188) = 2.09, p = 0.038). About two thirds of the individuals in the sample expressed a treatment goal of controlled drinking rather than abstinence. A slightly higher proportion of women than men (41% and 35% respectively, *X*^2^ (1, N = 189) = 0.63, p = 0.427) were screening positive for MDE or GAD. During the 12 months preceding their first contact with the Alcohol Helpline three out of four participants had been in contact with various health care services, but less than half had sought help for alcohol problems. At follow-up, respondents had significantly reduced their AUDIT score to half of the baseline values, indicating a decreased alcohol use and/or fewer alcohol problems. The decrease was evident both in terms of the AUDIT total score and the AUDIT zones. For instance, 10 percent of men and 11 percent of women had been abstinent, and 19 percent of the men and 23 percent of the women had used alcohol at low-risk levels. The proportion of men who were considered alcohol dependent had decreased from 68 to 18 percent, while the corresponding proportion among women decreased from 57 to 20 percent (Z = −10.23, p < 0.001). In addition, the reduction in AUDIT score was most prominent among individuals having higher scores at baseline (Figure 2). Of the participants originally classified as dependent only 27% remained in the same zone, compared to 57% of those with hazardous alcohol use (*X*^2^ (2, N = 189) = 7.10, p = 0.029).

**Table 2:** Alcohol-related outcomes and other clinical characteristics of the sample at baseline and follow-up, by gender

	**Men (n = 117)**			**Women (n = 74)**			**Total sample (n = 191)**		
	**Baseline**	**Follow-up**		**Baseline**	**Follow-up**		**Baseline**	**Follow-up**	
AUDIT score (Mean [sd])	22.9 (6.0)	11.8 (8.1)	<.001	21.0 (6.3)	10.4 (7.8)	.001	22.2 (6.2)	11.3 (8.0)	<.001
AUDIT zones (%)			<.001			.001			<.001
Abstinent	-	10		-	11			11	
Low-risk	-	19		-	23			21	
Hazardous	8	37		7	30		7	34	
Harmful	24	15		37	16		29	15	
Dependent	68	18		57	20		64	19	
Controlled drinking as treatment goal (%)	61			68			62		
MDE and/or GAD (%)	35	17	<.001	41	20	.002	38	18	<.001
Self-assessed health (very) good (%)	63	73	.099	53	80	<.001	59	75	<.001
Previous contact with health care (regardless of cause) (%)	71			81			75		
Help seeking for alcohol problems (%), past 12 months	47	60	.014	49	68	.016	47	63	<.001
In specialised addiction treatment	11	25	.002	7	24	.004	9	25	<.001
Only in other health care	15	14	1.000	10	12	.687	13	13	.265
Only other	21	21	1.000	32	31	1.000	25	25	.720
Number of sessions (Mean [sd])^#^	1.3 (0.5)	4.1 (4.6)		1.3 (0.6)	3.6 (3.8)		1.3 (0.6)	3.9 (4.3)	

^#^Due to frequent sessions with the Alcohol Helpline in the first phase of counselling, 24 men and 16 women had more than one session before the baseline interview was carried out within a few days after the first session.

Also the respondents’ mental health had improved significantly. The proportion meeting the criteria for MDE and/or GAD had been reduced by half. Moreover, the proportion of participants perceiving their health as good or very good had increased, significantly among women (Table 2).

It is noteworthy that a significantly higher proportion of both men and women had sought additional specialised addiction treatment for alcohol problems during the follow-up period than during the year preceding their first contact with the Alcohol Helpline. A similar change in AUDIT score was seen between those who had sought health care during the 12 months following the first contact and those who had not. The average reduction in AUDIT score was 10.2 points (sd: 8.1) for those who had sought help and 11.2 points (sd: 7.5) for the others (t (186) = 0.9, p = 0.391). Those who had sought help in health care had a higher AUDIT score at baseline (24.7, sd: 6.2) than the other participants (20.6, sd: 5.7, t(188) = 4.6, p < 0.001).

In Table 3 the odds of being in a lower AUDIT zone at 12-month follow-up for clients with repeated sessions relative to the odds for clients with only one session are presented, adjusted for the AUDIT total score at baseline. Among all respondents, the probability to belong to any lower AUDIT zone increased by 66 percent for individuals with two to three sessions and by 38 percent for individuals with four or more sessions as compared to those with just one session, but the estimates were not statistically significant.

**Table 3:** AUDIT change according to the number of counselling sessions at the Alcohol Helpline, by gender

	**Total sample (n = 188)**		**Excluding those who were abstinent at follow-up (n = 168)**	
**Total sample**	**Odds ratio**^**§**^	**95% CI**	**Odds ratio**^**§**^	**95% CI**
AUDIT score at baseline (continuous)	0.89	0.85-0.93***	0.86	0.82-0.90***
Number of sessions (1 session as reference)				
2-3 sessions	1.66	0.86-3.23	1.90	0.92-3.93
4 or more sessions	1.38	0.72-2.66	1.87	0.92-3.78
*Nagelkerke R*^*2*^	*15.2%*		*23.5%*	
**Men**	**(n = 114)**		**(n = 102)**	
AUDIT score at baseline (continuous)	0.86	0.81-0.91***	0.84	0.78-0.90***
Number of sessions (1 session as reference)				
2-3 sessions	1.08	0.45-2.58	1.29	0.49-3.39
4 or more sessions	0.76	0.32-1.79	1.14	0.45-2.86
*Nagelkerke R*^*2*^	*21.3%*		*26.8%*	
**Women**	**(n = 74)**		**(n = 66)**	
AUDIT score at baseline (continuous)	0.91	0.84-0.97**	0.86	0.79-0.94***
Number of sessions (1 session as reference)				
2-3 sessions	2.73	0.96-7.78	3.04	0.98-9.37
4 or more sessions	2.71	0.94-7.82	3.36	1.06-10.67*
*Nagelkerke R*^*2*^	*13.6%*		*24.2%*	

^§^Odds ratios indicate the odds of having an alcohol use pattern corresponding a lower AUDIT zone at follow-up, indicating a lower alcohol use or alcohol problems.

*Significant at p < 0.05; **p < 0.01; ***p < 0.001.

The test for interaction between gender and number of sessions was statistically significant (Wald Chi-square (1) = 4.1, p = 0.042 for 4 or more sessions), justifying separate gender analysis.

Women with more than one counselling session had a more than two-fold increased odds to belong to a lower AUDIT zone at follow-up compared to women with only one counselling session, while among men the odds ratios were close to one (Table 3).

After exclusion of participants who were abstinent at follow-up, women with more than one session had a significantly lower AUDIT score at follow-up than women with only one session, with a tendency to dose–response relationship (Table 3).

Associations were confirmed, albeit with lower precision, after adjustment for gender, age, mental health problems, treatment goal, help seeking for alcohol problems and the AUDIT score at baseline as potential confounders (Additional file 1). No significant interaction was found between the number of sessions at the Alcohol Helpline and reporting other help seeking during the study period.

## Discussion

This study presents a longitudinal analysis of clients of the Swedish National Alcohol Helpline, showing substantial reduction of self-reported alcohol use and alcohol problems over one year. These changes were observed despite that at the first contact with the helpline approximately two thirds of these clients had an AUDIT score indicating possible alcohol dependence. At this severity of alcohol problems the World Health Organization (WHO) recommends referral to specialists for diagnostic evaluation and treatment [30]. After 12 months nearly three-fourth of the participants had modified their alcohol use pattern to such an extent that they met the criteria for a lower AUDIT zone. Moreover, the whole group on average decreased their AUDIT score by half and one third of the participants were abstinent or consumed alcohol at a low-risk level. Further, among women there was an indication of dose-response effect with the intervention’s intensity.

There were also some important additional findings. Reported mental health problems were reduced by half and among women perceived health had improved significantly. Help seeking in specialised addiction treatment had increased. This was expected since referral to other service providers for clients in need of additional support is part of the intervention protocol at the Alcohol Helpline. However, data did not suggest any difference in outcome between clients only calling the Alcohol Helpline and those seeking other health care.

We are not aware of previous studies evaluating community based telephone counselling intended as a stand-alone intervention for self-referred hazardous and harmful alcohol users in the community. We find it interesting to contrast our findings to those of studies based on telephone counselling as part of multicomponent interventions for patients seeking health care for other reasons. In a study on psychiatric outpatients, a 15-minute telephone counselling was delivered to individuals with hazardous or harmful alcohol use by specially trained nurses. Results indicated that about 44 percent of patients had alcohol use at low-risk levels after six months [15], a finding very similar to ours, taking into account that patients with AUDIT scores indicating alcohol dependence were not included in that study. Twelve months after initial face-to-face physician advice and three follow-up telephone sessions with a health educator slightly less than half of older “at risk drinking” primary care patients had decreased their alcohol use to low-risk levels [16]. In a stepped care intervention among general practice patients with alcohol use disorders, initial assessment was followed by computerized feedback and up to four telephone counselling sessions delivered by trained psychologists. At 12-month follow up there was a significant reduction of alcohol consumption among patients with at-risk drinking or alcohol abuse but not among those with alcohol dependence or heavy episodic drinking [17].

Telephone counselling has frequently been used as part of extensive efforts to encourage patients first treated in specialised substance abuse treatment to attend aftercare or continuing care. In one of these studies alcohol-dependent individuals were assigned to three different conditions after attending a 4-week intensive out-patient treatment program. Participants in the telephone counselling condition that including one face-to-face contact showed significantly better outcomes than participants in the two face-to-face conditions [19]. Taken together, the findings of these studies are very much in line with those of the present study and support the conclusion that telephone based counselling is an effective way to tackle alcohol problems in the community.

In the present study we found that women with repeated counselling sessions tended to have a lower AUDIT score at follow-up compared to women with only one counselling session, especially when excluding women who were abstinent at follow-up. No hint of a dose-response relationship with counselling intensity was found among men. Albeit based on a limited sample, this observation is of interest, considering that some studies found limited gender differences in treatment outcome [7,20,21] while other studies show effectiveness of brief interventions among men compared to women [6,8]. It is therefore possible that behavioural changes among women require higher intensity of counselling compared to men, an opportunity available at low cost at the Alcohol Helpline.

It is important to recognise some limitations of this study when interpreting the results, particularly the study design. Since this was an observational study in a real world context, the absence of a control group makes it impossible to draw firm conclusions about a causal effect of counselling at the Alcohol Helpline in changing alcohol use among hazardous and harmful alcohol users. The observed behaviour change could be explained by factors that were not controlled for by the study, such as certain life events or client characteristics. However, the magnitude of the behavioural change, together with a suggestion of dose-response effect among women makes our statement about treatment effectiveness at least plausible.

Second, this study relies upon self-reported retrospective data, increasing the risk for information bias. The AUDIT covers alcohol use and alcohol problems during the previous 12 months, a relatively long period. Further, feelings of shame and guilt about own alcohol use may enhance the risk for socially desirable answers. However, the interview and counselling took place at different times, with interviewers blinded as to clients’ treatment, including the number of sessions. Selection of intervention intensity did not happen by chance, but was determined by a consideration of the clients’ wishes and needs, including the severity of the alcohol problems. However, confounding by indication would rather have biased the association between treatment and outcome toward an under-estimation of the intervention effect. In addition, we handled this risk by adjusting for baseline AUDIT and other potential predictors of outcome.

The external validity of the study might be questioned, as the study sample was not representative of all clients at the Alcohol Helpline or of the potential treatment seekers. For instance, individuals participating and retained in the study may have been particularly motivated to change their alcohol use. Therefore, caution is needed in generalizing these results or in establishing comparisons with other studies. However, we noted that participants’ AUDIT score, age and gender were comparable with those of the whole group of clients at the Alcohol Helpline during the same period.

Finally, the small sample size of the study entailed a low power to detect weak associations as statistically significant.

## Conclusions

To our knowledge, there is no qualified telephone counselling as a stand-alone intervention for hazardous and harmful alcohol users in the community, similar to the Swedish National Alcohol Helpline. Future randomized studies including other treatment or control conditions are warranted in order to strengthen our preliminary conclusion of possible effectiveness of the counselling provided at the helpline, as well as to explore further the role of gender in moderating the treatment’s effect.

There are some important clinical implications of this study. First, the Swedish National Alcohol Helpline provides a model for broadening the base of treatment for alcohol problems. Second, the reduction in alcohol use among clients of the Helpline was similar whether or not other supplementary care was sought, indicating high levels of behaviour modification. It should be borne in mind that help-seeking behaviour is an achievement per se, and this behaviour was enhanced among clients of the Helpline. Thus, a society with the ambition to reach problem drinkers at an early stage may consider telephone counselling in addition to a wide range of services to match needs and preferences in the target group.

## Abbreviations

AUDIT: Alcohol Use Disorders Identification Test; CES: Centre for Epidemiology and Community Medicine; GAD: Generalized anxiety disorder; MDE: Major depressive episode; MI: Motivational interviewing; MIC Lab: Motivational Interviewing Coding Laboratory; M.I.N.I.: M.I.N.I. International Neuropsychiatric Interview; MINT: Motivational Interviewing Network of Trainers; MITI: Motivational Interviewing Treatment Integrity; OR: Odds ratio; SCC: Stockholm County Council.

## Competing interests

KDT is head of the Swedish Alcohol Helpline operated by the CES in the SCC. NH is employed as a counsellor at the Swedish Alcohol Helpline. MT is employed at the CES. MRG holds a double affiliation at the CES and the Department of Public Health Sciences at the Karolinska Institutet.

## Authors’ contributions

MRG conceived the design of the study, supervised the data-analysis and the writing of the manuscript; NH carried out the data analysis and drafted the manuscript; MT supervised the data-analysis and KDT contributed the data material and supervised the writing of the manuscript. All authors substantially contributed to the interpretation and discussion of the results. All authors read and approved the final manuscript.

## Supplementary Material

Additional file 1 AUDIT change according to the number of counselling sessions at the Alcohol Helpline adjusted for sociodemographic and clinical characteristics, by gender. Click here for file
