# Supplementary material for: An evaluation of long-term changes in alcohol use and alcohol problems among clients of the Swedish National Alcohol Helpline
Source: Subst Abuse Treat Prev Policy. 2014 Jun 3;9:22. doi: 10.1186/1747-597X-9-22 (PMC4055694; doi:10.1186/1747-597X-9-22)
Supplement: Additional file 1 — AUDIT change according to the number of counselling sessions at the Alcohol Helpline adjusted for sociodemographic and clinical characteristics, by gender. [file 1747-597X-9-22-S1.docx]

|  | **Men (n=113)** | | **Women (n=73)** | | **Total sample (n=186)** | |
| --- | --- | --- | --- | --- | --- | --- |
|  | **Odds ratio §** | **95% CI** | **Odds ratio §** | **95% CI** | **Odds ratio §** | **95% CI** |
| **Total group (n=188)** |  |  |  |  |  |  |
| AUDIT score at baseline (continuous) | 0.84 | 0.78-0.90*** | 0.90 | 0.82-1.00* | 0.87 | 0.83-0.92*** |
| Controlled drinking as treatment goal | 0.47 | 0.21-1.06 | 0.51 | 0.19-1.37 | 0.46 | 0.25-0.83* |
| MDE and/or GAD | 1.79 | 0.80-4.03 | 1.34 | 0.43-4.18 | 1.78 | 0.95-3.33 |
| Help seeking for alcohol-related problems | 0.68 | 0.28-1.61 | 0.34 | 0.11-1.06 | 0.49 | 0.25-0.95* |
| Gender |  |  |  |  | 0.83 | 0.47-1.47 |
| Age (continuous) | 1.04 | 1.01-1.06* | 0.98 | 0.95-1.02 | 1.01 | 0.99-1.03 |
| Number of sessions (1 session as reference) |  |  |  |  |  |  |
| 2-3 sessions | 1.37 | 0.54-3.46 | 2.53 | 0.82-7.87 | 1.75 | 0.87-3.51 |
| 4 or more sessions | 0.79 | 0.32-1.97 | 1.99 | 0.64-6.14 | 1.25 | 0.64-2.46 |
| *Nagelkerke R^2^* | *31.7%* |  | *19.3%* |  | *22.2%* |  |

§ Odds ratios indicate the odds of having an alcohol use pattern corresponding a lower AUDIT zone at follow-up, indicating a lower alcohol use or alcohol problems.
* Significant at p <.05; *** p< .001
